# The Effect of Melatonin on Maturation, Glutathione Level and Expression of H MGB1 Gene in Brilliant Cresyl Blue (BCB) Stained Immature Oocyte

**Published:** 2013-11-20

**Authors:** Maryam Salimi, Mohammad Salehi, Reza Masteri Farahani, Maryam Dehghani, Mohammad Abadi, Marefat Ghaffari Novin, Mohsen Nourozian, Ahmad Hosseini

**Affiliations:** 1 Department of Biology and Anatomical Sciences, Faculty of Medicine, Shahid Beheshti University of Medical Sciences, Tehran, Iran; 2 Cellular and Molecular Biology Research Center, Shahid Beheshti University of Medical Sciences, Tehran, Iran; 3 Department of Biotechnology, Faculty of Medicine, Shahid Beheshti University of Medical Sciences, Tehran, Iran; 4 Department of Transgenic Animal Science, Stem Cell Technology Research Center, Tehran, Iran

**Keywords:** Melatonin, Glutathione, Oocyte, Brilliant Cresyl Blue Staining, *HMGB1*

## Abstract

**Objective:**

Nutrients and antioxidants in the medium of immature oocyte have a profound effect on maturation, fertilization and development of resulting embryos. In this study the effects of melatonin as an antioxidant agent on maturation, glutathione level and expression of *High mobility group box-1* (*HMGB1*) gene were evaluated in immature oocytes of mice stained with brilliant cresyl blue (BCB).

**Materials and Methods:**

In this experimental study, immature oocytes were harvested from ovaries of Naval Medical Research Institute (NMRI) mice. Oocytes were stained with 26 μM BCB for 90 minutes and transferred to *in vitro* maturation medium containing varying doses of melatonin (10-12, 10-9, 10-6, 10-3 M) and without melatonin, for 22-24 hours. Maturation was monitored using an inverted microscope. Glutathione was assessed by monochlorobimane (MCB) staining and *HMGB1* expression in mature oocyte was analyzed using real-time polymerase chain reaction (PCR).

**Results:**

Melatonin in the concentration of 10-6 M had the most effect on maturation and *HMGB1* expression of BCB+ oocytes (p<0.05). Meanwhile melatonin had no effects on glutathione levels. Additionally in immature BCB- oocytes, compared to the control group, melatonin did not affect cytoplasm maturation (p>0.05).

**Conclusion:**

*In vitro* treatment with melatonin increases the maturation and *HMGB1* expression in BCB+ immature oocytes and has no significant effect on glutathione levels.

## Introduction

Although the quality of *in vitro* maturation (IVM) is less than *in vivo* matured oocyte (1), it is a frequent technique in *in vitro* fertilization (IVF) centres for augmenting the number of mature oocyte for IVF. Maturation is defined in two parts of an oocyte: nuclear maturation visualized by the extrusion of the second polar body and cytoplasm maturation (2). Successful maturation, fertilization and development prior to implantation are dependent on growth and differentiation of immature oocytes and the surrounding cumulus cells. The two major factors affecting embryo production and development are the quality of immature oocyte and the composition of IVM medium (3). The first important step in production of *in vitro* embryos is selecting high quality oocytes in order to transfer to IVM medium to achieve mature oocytes (4). Generally for such selections few morphological criteria are used including the number of surrounding layers of cumulus cells, cytoplasm homogeneity, follicle and immature oocyte size (5) but they are inconsistent and unreliable (6). Brilliant cresyl blue (BCB) staining is a non-invasive method used for the selection of immature oocyte in animal studies and is related to increased maturation of embryos (7). BCB is a biomarker of glucose-6-phosphate dehydrogenase (G6PD) level in immature oocytes and has the highest expression in good quality oocytes and decreases with maturation (7).

*In vitro* maturation medium is a vital and effective factor in nuclear maturation, cleavage and embryo maturation, and blastocyst formation (3). In various studies amino acids and antioxidants have been used for oocyte maturation (8). Very low or high concentrations of free radicals in medium affects the maturation and cleavage of embryos *in vitro* (9). Studies used enzymatic antioxidants to regulate the levels of free radicals, such as catalase, turine and hypoturine (10). Melatonin is a tryptophan derived hormone secreted from pineal gland into oviduct and follicular fluid during ovulation (11). Therefore it exerts an important effect in the reproductive system (12). On the other hand, being an antioxidant, it has a role in scavenging the reactive oxygen species (ROS) in the environment (13). Its positive effects on embryo development through to blastocyst formation have been confirmed (14,15). As noted one of the processes in IVM is cytoplasm maturation encompassing biochemical molecules such as glutathione, phosphorylated proteins and the activation of metabolic pathways. Glutathione production is an important biomarker of cytoplasm maturation in IVM (16). In addition to its antioxidant property, glutathione is important in formation and stabilization of mitotic spindle assembly in mature oocyte and also in the formation of the male pronucleus (17). Studies have shown that higher concentration of glutathione in the cytoplasm is related to higher percentage of IVF success and maturation to blastocyst (18,19).

*High mobility group box-1* (*HMGB1*) gene has various functions including transcription, DNA repair (20) and apoptosis (21). Its product, *HMGB1* protein is expressed on the blastomere membrane throughout the maturation process. It has been shown that embryos expressing this protein reach the blastocyst stage in higher numbers (22). Melatonin as an antioxidant could have the potential for inductionor suppression of *HMGB1*.

Positive effects of melatonin on embryo development through to blastocyst formation have been confirmed although its effective dose in oocyte maturation of BCB stained oocyte remains unknown. So the aim of this study was to evaluate the effects of melatonin in IVM medium on glutathione level, *HMGB1* expression and maturation of immature oocyte stained with BCB.

## Materials and Methods

All experiments and protocols were performed in strict accordance with the guiding principles for the care and use of research animals adopted by the Shahid Beheshti University of Medical Sciences.

All chemicals were purchased from Sigma Chemical Corporation (St. Louis, MO, USA) except where noted otherwise.

### Oocyte collection

In this experimental study, oocytes were obtained from female Naval Medical Research Institute (NMRI) mice (Pasteur Institute, Iran) with age 6-8 weeks that were kept under controlled light and temperature conditions with free access to water and food. They had 12 hours light and 12 hours dark conditions. Mice were primed with 10 IU of pregnant mare serum gonadotropin (PMSG). The ovaries were removed 48 hours later and placed in tissue cell culture medium (TCM) 199 Hepes supplemented with 5% foetal bovine serum (FBS). The germinal vesicle (GV) stage oocytes were released by puncturing ovarian follicles with 28G needle.

### Brilliant cresyl blue staining

The cumulus-oocyte complexes (COCs) obtained were washed three times in flushingholding medium (FHM) and then incubated in potassium simplex optimized medium (KSOM) supplemented with 4% bovine serum albumin (BSA) containing 26 μM BCB for 90 minutes at 37˚C in humidified air atmosphere. After the incubation time, the oocytes were observed under microscope and classified according to BCB staining as i. dark blue cytoplasm (BCB+) and ii. colourless cytoplasm (BCB–) (23).

### *In vitro* maturation

Each group was placed in 50 μL microdrops of TCM-199 supplemented with 10% FBS, 0.2 mM sodium pyruvate, 2 mM L-Glutamin,10 μg/ mL follicle stimulating hormone (FSH), 10 μg/ mL luteinizing hormone (LH) and 1 μg/mL estradiol -17β with the additional of variety of concentration of melatonin (0, Dimethyl sulfoxide (DMSO), 10^-3^, 10^-6^, 10^-9^, 10^-12^ M) in a humidified atmosphere with 5% CO_2_ at 37˚C for 22-24 hours. COCs showing fully expanded cumulus cells after 24 hours maturation period, were assessed by phase contrast inverted microscope (Olympus, Japan) and COCs which were not expanded or showed incomplete expansion were not accounted (24).

### Monochlorobimane staining

To estimate the glutathione concentration in oocytes we used a fluorescent indicator of glutathione, monochlorobimane (MCB) (25). Oocytes were incubated with 50 mM MCB in FHM medium for 45 minutes and then fluorescence of MCB was recorded at 390 nm by a digital camera. Intensity of fluorescence was analyzed by Image J software (National Institutes of Health, Bethesda, MD, USA).

### Relative expression of high mobility group box-1

Relative amounts of *HMGB1* gene transcripts were determined by using a real time PCR (Polymerase chain reaction). At least 10 oocytes were analyzed for each group andtransferred to the bottom of a 0.2 mL Eppendorf tube containing 1.5 μL lysis buffers (26) and processed for reverse transcription-polymerase chain reaction (RT-PCR). All RT-PCR solutions were prepared in Milli-Q Ultrapure water. Two microliters of poly N and 5 μL water were added to embryo and placed in thermocycler for 5 minutes 75˚C. After, the tubes were placed on ice and 9 μL of the following reaction mixture (5x RT Buffer, 200 u RT Enzyme, 10 mM dNTP and 10 u RNase inhibitor) were added to embryo samples. Both RT-PCR and PCR reactions were performed on an applied Bio Rad thermocycler. The ampliﬁcation program for the reverse transcription step was as follows: 25˚C for 10 minutes, 37˚C for 15 minutes, 42˚C for 45 minutes and 72˚C for 10 minutes.

After the reverse transcriptase reaction, samples were kept at 4˚C overnight; then to each sample, PCR mixtures were added: 1.25 μL Taq Polymerase, 20.75 μL Master Mix, 2 μL cDNA and 2 μL speciﬁc primers. The endogenous control *Hprt1* (F: TCCCAGCGTCGTGATTAG, R: CGAGCAAGTCTTTCAGTCC, Accessionno: NM_013556.2) and *HMGB1* (F: GAAGTATGAGAAGGATATTGCTG, R: CCAACTTATTCATCATCATCATC, Accession no: NM_010439.3) genes were ampliﬁed with the following PCR cycle programme: 94˚C for 3 minutes, 60˚C for 45 seconds, 72˚C for 45 seconds for 40 cycles followed by 72˚C for 7 minutes. Ten microliters of PCR product were mixed with 1 μL loading buffer and were electrophoresed on a 2% agarose gel in Tris-acetate- EDTA (TAE) buffer for 25 minutes. The products were visualised under short-wave length Ultraviolet (UV).

Real-time quantitative PCR was performed to assess the expression of *HMGB1* gene using Rotor Gene Q instrument (QIAGEN). Real time PCR reactions were carried out in a total volume of 13 μL according to the manuals for DNA Master SYBR Green I mix (Roche Applied Sciences). The primer concentrations were adjusted to 1 μM for each gene. The cycling parameters were 5 seconds at 95˚C, 3 minutes at 95˚C for denaturation, 15 seconds at 60˚C, 10 seconds at 72˚C for ampliﬁcation and extension respectively for 40 cycles. The speciﬁcity of all individual ampliﬁcation reactions was conﬁrmed by melting curve analysis. The assays used *Hprt1* as the endogenous internal house-keeping gene. Three replications were performed and the mRNA level of each sample was normalized to that of *Hprt1* mRNA level. The relative levels of mRNA were analyzed by the REST 2009 Software (QIAGEN).

### Statistical analysis

All statistical analysis was performed using Service Provisioning System Software (SPSS) 16 for windows (SPSS, Chicago, IL, USA). The means of metaphase II (MII), cumulus expansion and metaphase I (MI), were compared by non-parametric analysis test (Kruskal-Wallis). Glutathione levels in experimental groups were compared by Analysis of Variance (ANOVA). Data are expressed as means ± SD. A statistically significant difference was accepted at p<0.05.

## Results

### Oocyte maturation

**Fig 1:**
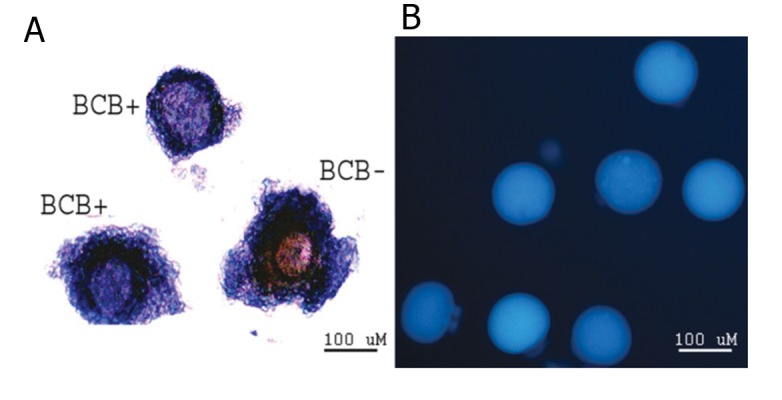
Immature oocytes stained with BCB, oocyte with blue coloration of cytoplasm (BCB+) and without blue cytoplasm (BCB-) (A). Fluorescent intensity of MCB stained mature oocyte (B).

1238 immature BCB+ oocyte were transferred to IVM media containing varying concentrations of melatonin (10^-12^, 10^9^, 10^-6^, 10^-3^ M, control and DMSO) (Fig 1A). It was observed that compared to the control group and other groups the number of metaphase II oocytes were significantly higher in media supplemented with 10^-6^ M of melatonin (66 and 76% respectively). However in low concentrations of melatonin (10^-12^ and 10^-9^) it had a negative effect on nuclear maturation (41 and 45% respectively). The highest degree of cumulus expansion was seen in the control group (92%) which only had a significant difference with 10^-12^ M of melatonin group. Metaphase I arrest was seen mostly in oocytes treated with 10^-9^ and 10^-12^ M of melatonin (54 and 58% respectively) as compared with the control (31.5%, p<0.05, Table 1).

**Table 1:** Development of BCB+ immature oocyte cultured in the maturation medium supplemented with different concentrations of melatonin


Group	No. oocyte	MI	Cumulus expansion	MII

**Control**	204	64(31.46 ± 3.41) ^a^^,^^b^	187(91.62 ± 1.25)^e^	136(66.69 ± 3.26) ^f^^,^^g^^,^^h^
**DMSO**	203	83(40.76 ± 5.25)	164(81.04 ± 2.35)	120(59.24 ± 5.25)
**Melatonin 10^-^^3^M**	198	93 (46.56 ± 23.27)	131(66.24 ± 15.46)	105(52.73 ± 20.7)
**Melatonin 10^-^^6^M**	188	44(23.63 ± 12.29) ^c^^,^^d^	163(87.1 ± 7.22)	144(76.37 ± 12.29) ^f^^,^^i^^,^^k^
**Melatonin 10^-^^9^M**	201	109(54.13 ± 16.98 )^a^^,^^c^	161(80.17 ± 14.29)	92(45.87 ± 16.98)^g^^,^^i^
**Melatonin 10^-^^1^^2^M**	206	121(58.90 ± 16.55) ^b^^,^^d^	130(63.15 ± 15.41)^e^	85(41.1 ± 16.55) ^h^^,^^k^


Within the same column, values with same letters were significantly different (p <0.05).

Additionally, 334 immature BCB- oocytes were transferred to media containing varying concentrations of melatonin (Fig 1A). The percentage of metaphase II oocytes and cumulus expansion was similar to the concentration of 10^-3^ M of melatonin (control: 35 and 73%; 10^-3^ M: 45 and 88% respectively). The majority of metaphase I arrest were seen in 10^-12^ M (82%) and 10^-9^ M (83%) which had a significant difference with control and 10^-3^ M group (60%, Table 2). According to tables 1 and 2 the BCB+ oocytes had a greater expansion and metaphase II oocytes and a lower percentage of metaphase I arrest compared to BCB- counterparts.

### Glutathione level in oocytes

**Fig 2:**
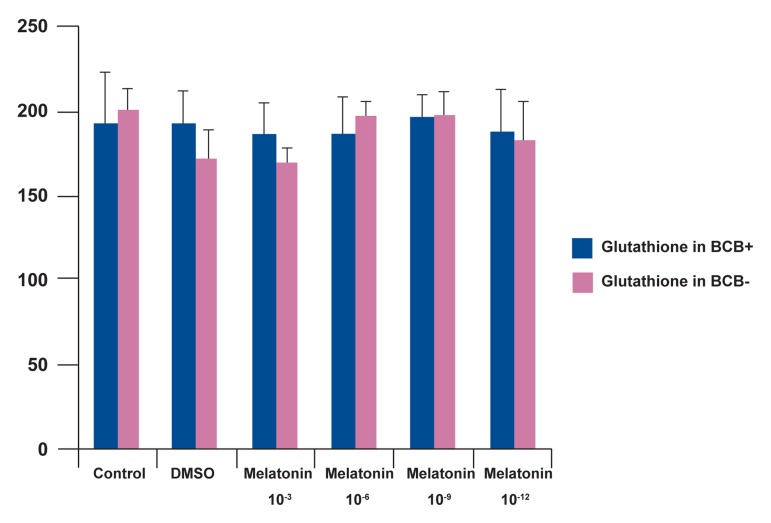
Glutathione level of BCB+ and BCB- oocytes cultured in different concentrations of melatonin. The Glutathione level was evaluated with MCB staining.

Our results showed that melatonin had no significant effect on the level of glutathione in oocytes (Figs 1B and 2).

### *HMGB1* expression level

**Fig 3:**
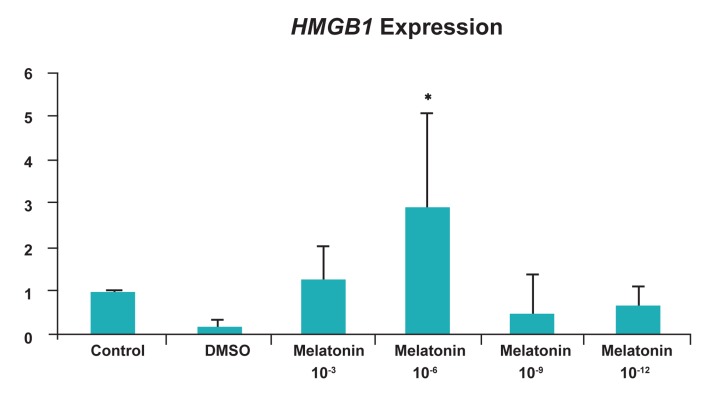
Relative expression levels of HMGB1 gene in BCB+ oocytes cultured with different concentrations of melatonin and without melatonin. The mRNA levels of HMGB1 were analyzed with real-time PCR and mRNA levels were normalized to that of Hprt mRNA level. *; p<0.05.

The expression of *HMGB1* gene was analyzed using real time-PCR in BCB+ oocytes. As seen in figure 3, *HMGB1* expression was at its highest in 10-6 M of melatonin compared to the control group and also had the lowest level in DMSO treated group (p<0.05) (Fig 3).

**Table 2:** Development of BCB- immature oocyte cultured in the maturation medium supplemented with different concentrations of melatonin


Group	No. 0ocyte	MI	Cumulus Expansion	MII

**Control**	54	32(60.07 ± 11.52)^a^^,^^b^^,^^c^	40(73.41 ± 20.36)^d^^,^^e^^,^^f^	21(38.25 ± 11.65)^g^^,^^h^^,^^i^
**DMSO**	62	45(73.18 ± 11.67)	31(50.9 ± 12.19)^d^	17(26.82 ± 11.67)
**Melatonin 10^-^^3^M**	57	34(59.95 ± 19.97)	50(88.28 ± 14.59)	26(45.82 ± 15.77)
**Melatonin 10^-^^6^M**	64	53(82.17 ± 7.50)^a^	22(35.20 ± 11.97)^e^	11(17.82 ± 7.5)^g^
**Melatonin 10^-^^9^M**	52	43(83.47 ± 11.16)^b^	21(40.1 ± 14.3)^f^	9 (16.52 ± 11.17)^h^
**Melatonin 10^-^^1^^2^M**	62	54(87.32 ± 12.28 )^c^	32(51.81 ± 18.73)	8(12.68 ± 12.28)^i^


Within the same column, values with same letters were significantly different (p <0.05).

## Discussion

Due to the beneficial uses of BCB staining we used the same protocol for the selection of immature oocytes and results showed that maturation was higher in BCB+ compared to BCB- oocytes which is in accordance with previous studies (7,23). We also compared the effects of the antioxidant melatonin on the maturation of oocytes. Among the most important harmful factors affecting oocyte and embryo are free radicals. They have deteriorating effects on DNA repair, mitotic spindle assembly and maturation of oocyte (9). Studies have utilized various enzymatic antioxidants such as catalase and non-enzymatic antioxidants including thioredoxin pyruvate and glutathione (27). Melatonin (N-acetyl 5-metoxy tryptamin) is a hormone (11) and its role as an antioxidant in the reproductive system was revealed when its levels in the follicular fluid and its receptors on granulosa cells and reproductive organs such as ovary, testis and fallopian tube were discovered. Melatonin stimulates progesterone secretion and suppresses the production of prostaglandins (28). Additionally it has the ability to stimulate the expression a number of antioxidant enzymes (13). Also it has anti-apoptotic potentials on various cells and embryos (24,29). However Takada et al. (30) have reported that melatonin in maturation medium fails to improve oocyte maturation, embryo development rates and DNA damage of bovine embryos. The results of this study showed that the optimum concentration of melatonin in IVM medium is 10-6 M and very low and very high doses have negative effects. Thus as ROS in a controlled concentration is vital for oocyte maturation, very high and very low doses can be detrimental for oocyte during IVM. Therefore the concentration of antioxidants becomes crucial as shown by others (14,24,29). However melatonin had no effect on the maturation of BCB- oocytes in this study.

*HMGB1* expression reduces blastocyst apoptosis and subsequently increases survival and development of embryos by suppressing p53 signalling and the expression of apoptosis-related genes Casp3 and Bax (31). *HMGB1* expression varies in different tissues in response to ROS and antioxidants for example antioxidants reduce its expression in pancreas and oxidants induce its expression in lymphatic tissues specially monocytes and macrophages (31). By suppressing DNA methyl transferase (DNMT), melatonin increases the expression of genes effective in embryo development (32). In the study of Cui et al. (22) it was observed that the expression of *HMGB1* before implantation, was highest inzygote, low in two cell stage and increases inmorulla and blastocyst and also higher levels attribute to maturation, expansion and successive stages of embryo development. Our study, for the first time, revealed that the expression of *HMGB1* increases in oocytes treated with 10-6 M concentration of melatonin in IVM medium. It was assumed that melatonin with its antioxidant activity could down regulate the expression of *HMGB1*, however by suppressing DNA methyl transferase, melatonin increases the transcription of genes involved in early maturation of embryos such as *HMGB1*. A possible mechanism involved in increased maturation and development of embryos by melatonin, could be the enhanced expression of *HMGB1* which in turn increases the transcription and DNA repair processes and oocyte maturation and subsequent development of embryos.

Somatic cells and gametes possess high amounts of glutathione which have an important role in oocyte maturation, fertilization and development prior to implantation as its presence in the semen proves its protective role (33). Production of glutathione during oocyte maturation has a profound impact on fertilization and embryo development (17). In oocyte, glutathione stabilises the mitotic spindle against oxidizing agents and is involved in enhancement of metaphase II, normal formation of egg, male pronucleus formation and inhibition of two cell stage arrests (34). The production of glutathione in IVM is influenced by the presence of various thiol amino acids or beta mercaptoethanol (35). In this study melatonin as an antioxidant and DNMT inhibitor was added to oocyte IVM medium to evaluate its effect on cytoplasm maturation. The results revealed that although melatonin increases nuclear maturation it is ineffective on glutathione and cytoplasm maturation. Therefore its effective properties are exerted at the nuclear level by increasing the expression of genes involved in oocyte maturation. Thus for achieving a better maturation it is best that in addition to melatonin other factors with the ability to enhance cytoplasm maturation be considered.

## Conclusion

The present study shows that *in vitro* treatment with melatonin increases the maturation and *HMGB1* expression in BCB+ immature oocytes and has no significant effect on glutathione levels.
